# Decoding the Histomorphological and Multi-Omic Characteristics of Melanotic Schwannoma

**DOI:** 10.1007/s11596-026-00197-6

**Published:** 2026-04-17

**Authors:** Yi Jiang, Rui Zhao, Hang Xian, Lin Shi, Jin-kang Zhang, Song-lin Li, Ye Peng, Jun-jie Du, Rui Cong, Han Wang

**Affiliations:** 1 Department of Neurology, Xi’an Central Hospital, Xi’an, 710004 China; 2 Department of Orthopedics, The First Affiliated Hospital of Air Force Medical University (Xijing Hospital), Xi’an, 710032 China; 3 Department of Orthopedics, Air Force Medical Center, Beijing, 100142 China

**Keywords:** Melanotic schwannoma, Multi-omics, BTK inhibitor, Neuropathic pain, Targeted therapy, Transcriptomic analysis, Proteomic analysis, Tyrosinase-related protein 1 (TYRP1)

## Abstract

**Objective:**

Melanotic schwannoma (MS) is a rare peripheral nerve sheath tumor accompanied by melanin deposition and severe refractory pain. However, its molecular pathogenesis remains unclear. This study aimed to explore the histomorphological, transcriptomic, and proteomic characteristics of MS, and to identify key molecules related to melanin production, pain generation, and targeted therapy.

**Methods:**

Histomorphological characteristics of MS were observed by Luxol Fast Blue (LFB) staining and hematoxylin and eosin (HE) staining in tumor tissues from 3 MS patients. Combined transcriptomic and proteomic analyses were performed to screen differentially expressed genes and proteins. The expression levels of tyrosinase-related protein 1 (TYRP1), transient receptor potential ankyrin 1 (TRPA1), and Bruton’s tyrosine kinase (BTK) were validated by quantitative real-time polymerase chain reaction (qRT-PCR), Western blotting, and immunofluorescence staining.

**Results:**

Histomorphological examination revealed typical melanin deposition and prominent compression of adjacent nerve fibers in MS. Transcriptomic and proteomic analyses showed activation of the melanin synthesis pathway and transient receptor potential (TRP) channel-related neuroinflammatory pathway. The expression of TYRP1 and TRPA1 was significantly upregulated, suggesting that these related pathways are associated with melanin production and neuropathic pain in MS. In addition, the expression of BTK, a known molecular target of targeted drugs, was significantly increased, suggesting that BTK inhibitors have potential therapeutic effects on MS.

**Conclusion:**

This study delineates the histomorphological features, transcriptomic and proteomic profiles of MS. TYRP1, TRPA1, and BTK are identified as key molecules involved in melanogenesis, pain generation, and targeted therapy, respectively. These findings provide novel insights into the pathogenesis and potential therapeutic strategies for MS.

**Supplementary Information:**

The online version contains supplementary material available at 10.1007/s11596-026-00197-6.

## Introduction

Melanotic schwannoma (MS) is a rare tumor of the nervous system that occurs mainly in the spinal and cranial nerve roots. It has also been reported in sympathetic ganglia and gastrointestinal nerve system, but it is extremely rare in peripheral nerves [1]. MS was first reported in 1932 as a malignant lesion in the thoracic sympathetic ganglia [2]. Since then, only approximately 200 cases of MS have been reported worldwide. The cellular origin of MS remains unclear. Some studies have reported that MS cells have the structural features of both Schwann cells and melanocytes, suggesting that they may be derived from their common stem and progenitor cells. Other studies have proposed that MS cells are derived from genetic mutations in Schwann cells [3–5]. Despite these findings, the detailed histopathological features, molecular mechanisms, and key pathogenic molecules of MS have not been fully characterized.

Given its extreme rarity, the biological behaviour and malignant potential of MS remain clinically unpredictable prior to pathological examination. With a metastatic rate of 13%–26%, the conventional view of MS as a benign tumour with rare malignant transformation may misguide the selection of treatment options. Furthermore, in many cases, advanced MS can cause severe refractory neuropathic pain that is difficult to manage [6–8]. Current evidence indicates that the efficacy of chemotherapy, radiotherapy or immunotherapy for MS is inconsistent [6–8]. Additional research is therefore needed to identify effective therapeutic targets to inhibit tumor growth and improve patient survival.

In this study, we performed histomorphological observation combined with transcriptomic and proteomic profiling to systematically characterize MS. We aimed to identify key molecules related to melanogenesis, neuropathic pain, and targeted therapy. The results may provide new insights into the histopathological and molecular features of MS, and lay a foundation for improving its clinical diagnosis and individualized treatment.

## Materials and Methods

### Samples

We obtained tumor tissues from 3 patients with MS who were admitted to the First Affiliated Hospital of the Air Force Military Medical University (AFMU), China. The patients’ general clinical characteristics were well-matched to meet the requirements of a matched-pair design. The specimens were collected upon obtaining approval from the Ethics Committee of the Air Force Military Medical University (Approval number: KY20232085-D-3, Date: 2023-03-07), and written informed consent was obtained from the patients or their legal family members. All the specimens were handled in accordance with the relevant ethical and legal guidelines. These clinical samples were used for transcriptome sequencing and immunostaining assays. Detailed information regarding the sources of tumor and normal tissues included in this study is provided in the supplementary materials.

### RNA Sequencing

Transcriptome analysis was performed in accordance with the methods described in a previous study [9], with the detailed procedures as follows. To assess sample purity, we used a NanoPhotometer® spectrophotometer (Thermo Fisher, USA) and an Agilent 2100 RNA Nano 6000 Assay Kit (Agilent Technologies, USA) to determine the integrity and concentration of RNA samples. Following confirmation of sample eligibility, mRNA was enriched using oligo(dT)-conjugated magnetic beads, after which fragmentation buffer was added to shear the mRNA into short fragments. First-strand cDNA was synthesized using six-base random primers with the fragmented mRNA as the template, and second-strand cDNA was subsequently generated by adding buffer, dNTPs, RNase H, and DNA polymerase I. The resulting cDNA was purified using a QIAQuick PCR Purification Kit and eluted with EB buffer. After elution and purification, the double-stranded cDNA was subjected to end repair, dA-tailing, and adaptor ligation, followed by adaptor-mediated PCR amplification. After the amplified products passed quality control, denaturation and single-strand cyclization were performed, and the cyclization products were validated. Qualified cyclized products were used to prepare DNA nanoballs (DNBs), which were then loaded onto an MGI platform sequencer for sequencing. The PE150 (paired-end 150 bp) sequencing strategy was employed for all samples. The raw RNA sequencing data have been submitted to the SRA database, and the BioProject accession number is PRJNA1083972.

### Proteome Analyses

Proteome analysis was performed in accordance with the methods described in a previous study [10], with minor modifications. MS tumor tissues were mechanically lysed in RIPA buffer supplemented with a protease inhibitor cocktail, and the supernatant was collected by centrifugation for protein quantification using a BCA assay. Equal amounts of protein from each sample were subjected to trichloroacetic acid (TCA) precipitation and acetone washing, followed by reconstitution in 200 mM TEAB and ultrasonic dispersion. Trypsin digestion was conducted overnight at a mass ratio of 1:50, with subsequent protein reduction (5 mM DTT) and alkylation (11 mM IAA, 15 min, dark). The digested peptides were desalted using a Strata X C18 column, lyophilized, reconstituted in 0.5 M TEAB, and labeled with a TMT kit according to the manufacturer’s instructions.

Peptide separation was performed on an EASY-nLC 1200 system with a mobile phase gradient elution (0–60 min), and peptides were ionized via a nanospray ionization (NSI) source and analyzed on a Q Exactive™ HF-X mass spectrometer in data-dependent acquisition (DDA) mode. The top 20 precursor peptides were selected for higher-energy collisional dissociation (HCD) with a collision energy of 28%. Raw mass spectrometry data were searched against the Homo sapiens protein database using the SEQUEST algorithm in Proteome Discoverer 2.5, with a precursor mass tolerance of 10 ppm and a fragment mass tolerance of 0.02 Da. Spectra with a mass range outside 350–5000 Da were excluded from the analysis. The mass spectrometry proteomics data have been deposited to the ProteomeXchange Consortium via the iProX partner repository [11, 12] with the dataset identifier PXD050541.

### Bioinformatics

Gene Ontology (GO) and Kyoto Encyclopedia of Genes and Genomes (KEGG) analyses were conducted on the Omicsbean platform (http://www.omicsbean.com:88/). MetaboAnalyst (http://www.metaboanalyst.ca/) and MetaGeneAlyse (http://metagenealyse.mpimp-golm.mpg.de/) were used to analyse the significant metabolites screened by KEGG metabolic pathway analysis and independent component analysis (ICA). Additionally, the integrated KEGG pathways were analysed using Pathview (https://pathview.uncc.edu/analysis).

### Luxol Fast Blue (LFB) Staining

The paraffin sections were sequentially immersed in xylene, anhydrous ethanol, different concentrations of alcohol, and distilled water. They were then dyed with 0.1% LFB at 60 °C for 8–16 h, rinsed with distilled water, and placed in 70% alcohol. The differentiation time was controlled through direct microscopic observation until the colour of the grey matter became lighter. The step was repeated if the differentiation effect was unsatisfactory. Afterwards, they were restained with 0.1% eosin for 1 min, rinsed again, and dehydrated with anhydrous ethanol and xylene. Finally, they were sealed with neutral gum. Microscopy was used to collect images, which were subsequently analysed.

### Hematoxylin and Eosin (HE) Staining

Paraffin sections were dewaxed, and sequentially dehydrated and cleared with methylene chloride and absolute ethanol, followed by a final rinse with distilled water. The sections were stained with haematoxylin solution for 3–8 min, differentiated with acid alcohol, rinsed with tap water, blued with ammonia water, and rinsed again with tap water. Next, the sections were stained with eosin for 1–3 min, dehydrated with 95% ethanol, and cleared with methylene chloride. The sections were air-dried slightly and mounted with neutral balsam. Finally, images were observed, collected and analysed using a microscope and associated software.

### Immunohistochemistry (IHC)

Tissue sections were dewaxed in xylene and rehydrated through a graded ethanol series. Endogenous peroxidase activity was blocked with 3% H_2_O_2_, and antigen retrieval was performed using microwave. After blocking with non-immune serum, primary antibodies were incubated with the sections, which were then washed with PBS. Biotin-conjugated secondary antibodies and streptavidin-peroxidase complex were added sequentially, with PBS washes between each incubation. Color development was achieved with DAB, followed by counterstaining and mounting with neutral balsam. The positive expression of target proteins was observed and quantified in 3 high-power fields (HPFs) per section. Information about the antibodies used in this study is provided in the Table S1.

### Immunofluorescence (IF) Staining

The paraffin-embedded sections were pretreated by dewaxing. The sections were permeated with 0.2% Triton X-100 for 5 min and blocked with serum from a secondary antibody source for 30 min. The antibody specific to the target antigen was selected as the primary antibody, which was diluted at a ratio of 1:100 and then added to the sample and incubated at 4 °C overnight to fully bind the primary antibody to the antigen. The samples were washed with PBS to remove unbound primary antibodies. The fluorescently labelled secondary antibody matching the primary antibody was selected, diluted at a ratio of 1:500, added to the sample, and incubated at room temperature for 2 h to bind the secondary and primary antibodies. Unbound secondary antibodies were removed by washing the samples with PBS, and the nuclei were stained with DAPI solution for 5 min. The samples were mounted in glycerol and stored at − 20 °C. The fluorescence signal was observed by fluorescence microscopy or laser confocal scanning microscopy, and the experimental results were recorded. Information about the antibodies used in this study is provided in the Table S1.

### Western Blotting

Total protein was extracted from MS tumor tissues using RIPA tissue lysis buffer. Protein concentration was detected using a BCA kit. After high-voltage electrophoresis, proteins were transferred to a PVDF membrane. The membrane was blocked with 5% non-fat milk. The membrane was incubated with primary antibodies overnight at 4 °C, followed by incubation with horseradish peroxidase-labeled secondary antibodies. Finally, protein bands were visualized using enhanced chemiluminescence (ECL) reagent and imaged. Information about the antibodies used in this study is provided in the Table S1.

### Quantitative Real-Time Polymerase Chain Reaction (qRT-PCR)

Total RNA was extracted from nerves and tumor tissues using TRIzol reagent. Total RNA (0.5 μg) was converted into cDNA through reverse transcription, and qRT-PCR amplification was performed according to the manufacturer’s instructions of the qRT-PCR kit. The reaction conditions were as follows: preheating at 95 °C for 30 s, denaturing at 95 °C for 5 s, and annealing at 60 °C for 20 s, for a total of 40 cycles. GAPDH was used as the internal control. Information about the primers used is provided in Table S2.

### Statistical Analysis

All analyses were repeated three times independently. The data are expressed as the mean ± standard deviation. For comparisons between two groups, a *t* test was conducted using SPSS 22.0 software. Differences with a *P* value < 0.05 were considered to be statistically significant.

## Results

### Histomorphological Features of MS

**Fig. 1:**
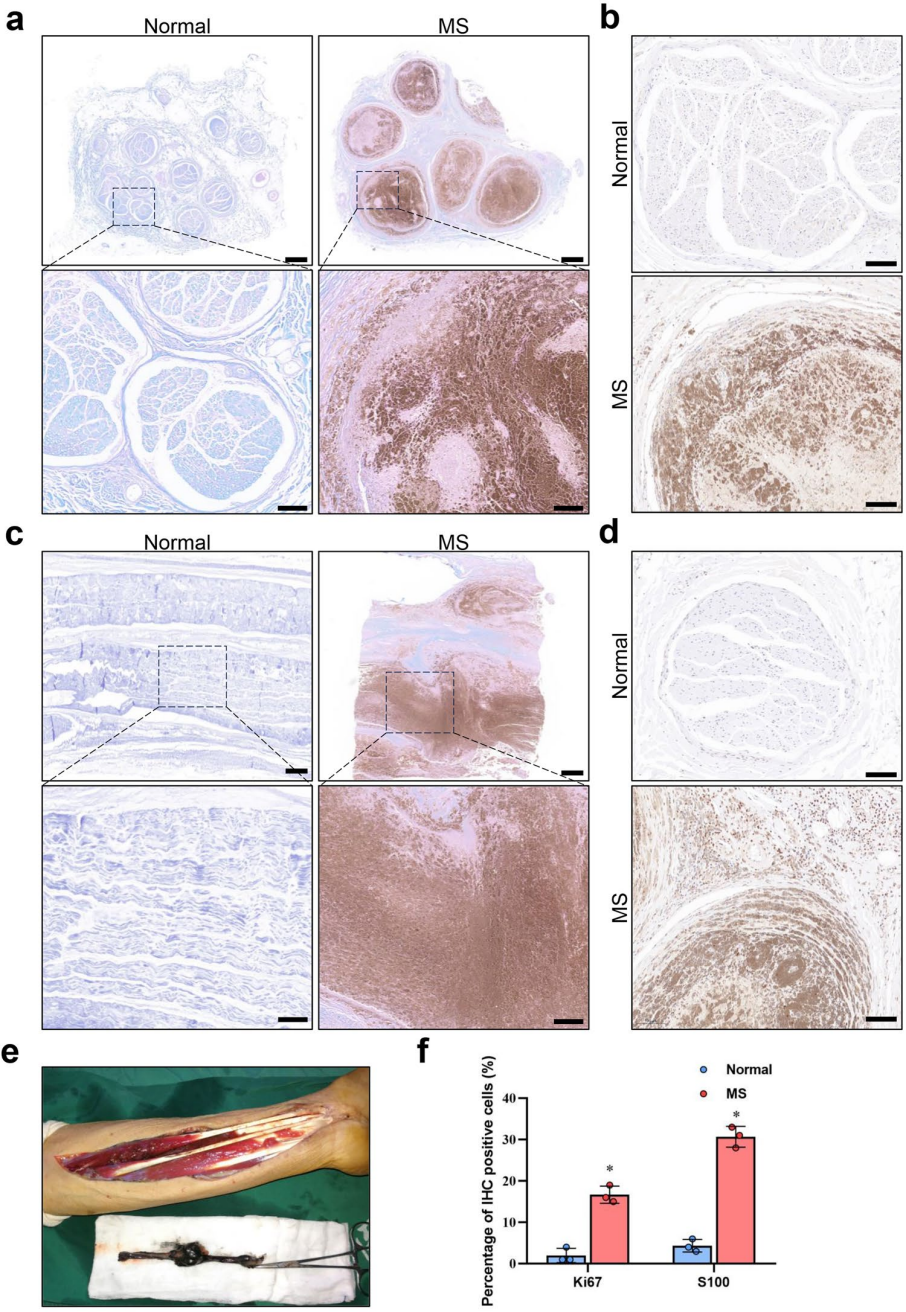
Histologic features of MS. LFB-stained cross-sections (**a**) and longitudinal sections (**c**) of MS and normal nerves. Scale bar = 500 μm (top) or 100 μm (bottom). IHC staining for Ki67 (**b**) and S100 (**d**) in MS and normal nerves. Scale bar = 100 μm. **e** Intraoperative image of a representative MS. **f** Quantification of Ki67^+^ and S100^+^ cells in MS and normal nerves. Data are presented as the means ± SD; ^*^P < 0.05 vs. normal nerves

Tumor tissues were derived from the peripheral nerves of MS patients, and histomorphological observation of these specimens revealed distinct morphological alterations in the affected peripheral nerve tissues with typical pathological manifestations of MS. LFB staining of cross-sections showed massive aggregated dark brown deposits distributed in patches within the perineurium of MS-invaded nerves, which replaced the normal nerve fiber structure; scattered dark brown deposits were also detected in the connective tissue between the perineurium and epineurium (Fig. 1a). Longitudinal LFB staining demonstrated irregularly arranged MS cells that almost filled the perineurial space, with the continuous nerve fiber structure observed in normal nerves being absent (Fig. 1c). IHC confirmed that S100-positive cells accounted for 30.67% of tumor tissues, further validating the diagnosis of MS; in contrast, the Ki67-positive rate reached 16.67%, indicating robust proliferative activity and malignant potential of the tumor (Fig. 1b, d, f). Intraoperative observations identified unique morphological changes of peripheral nerve MS, including partial or complete blackening of nerve bundles, nodular masses on nerve bundles, and black exudates (Fig. 1e).

**Fig. 2:**
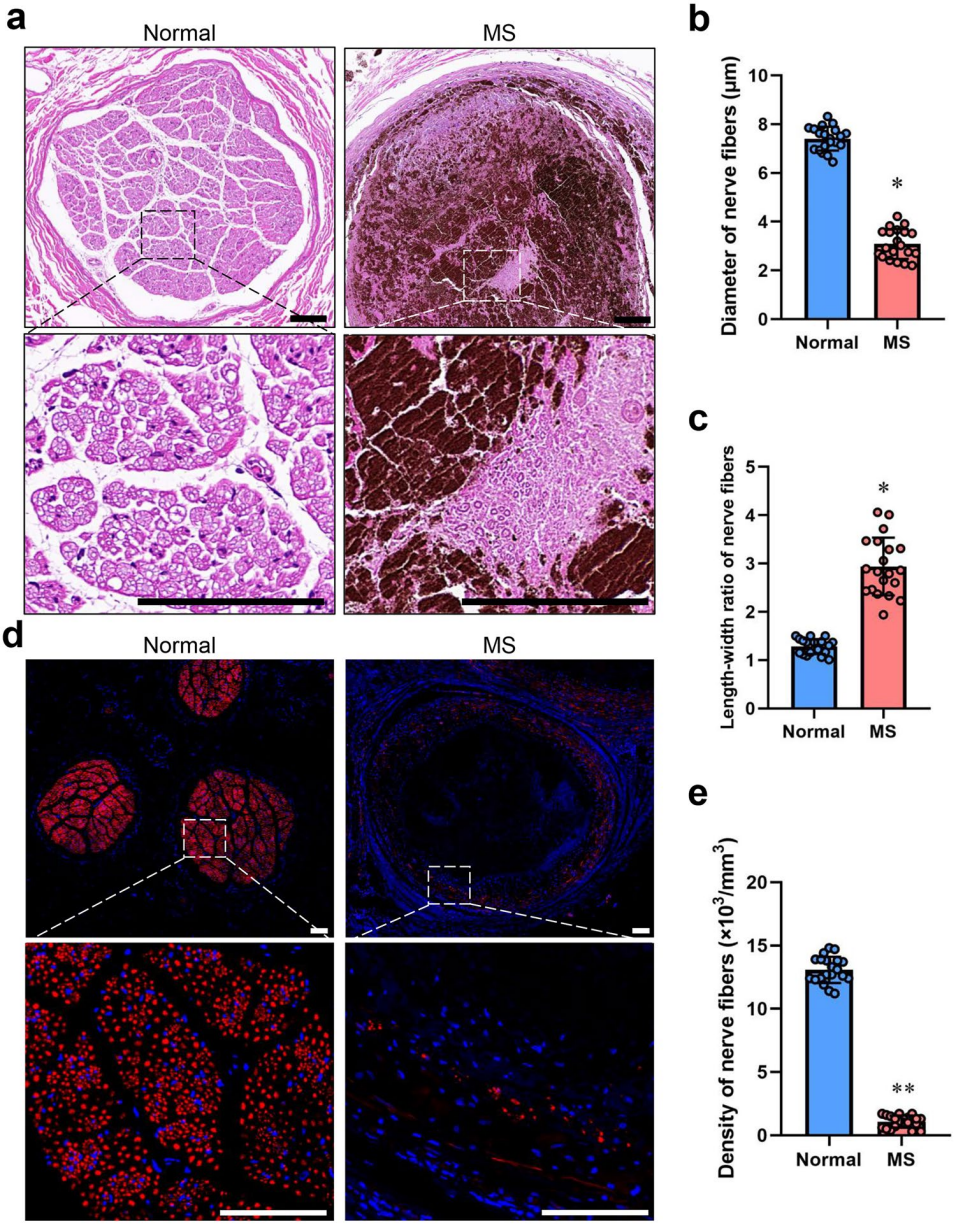
Histological changes in nerve fibres in MS. **a** HE-stained cross-sections of MS and normal nerves. Scale bar = 500 μm (top) or 100 μm (bottom). **b, c** Quantification of nerve fibre diameter (**b**) and length–width ratio of nerve fibre (**c**) in MS and normal nerves. **d** β3-Tubulin IF staining of MS and normal nerves. Scale bar = 100 μm. **e** Quantification of the nerve fibre density in MS and normal nerves. The results are shown as the mean ± SD; ^*^P < 0.05, ^**^P < 0.01 vs. normal nerves

High-magnification HE staining displayed well-defined nerve fiber and myelin sheath structures in normal nerve tissues (Fig. 2a). In the MS group, a large number of black deposits in nerve bundles replaced the original nerve fibers, and the remaining few nerve fibers were compressed and deviated from their normal morphological structure. Compared with the normal group, MS group exhibited significantly reduced nerve fiber diameter and an increased ratio of long to short diameter (Fig. 2b, c), suggesting obvious compression and narrowing of nerve fibers in MS. β3-Tubulin IF staining further showed that the density of nerve fibers in MS nerve bundles was markedly lower than that in the normal group, with only a small number of nerve fibers distributed around nerve bundles and in the inner layer of the perineurium (Fig. 2d, e). Additionally, no nuclei were found in the central area of MS nerve bundles, implying tumor cell necrosis in this region.

### Transcriptomic Analysis of MS

**Fig. 3:**
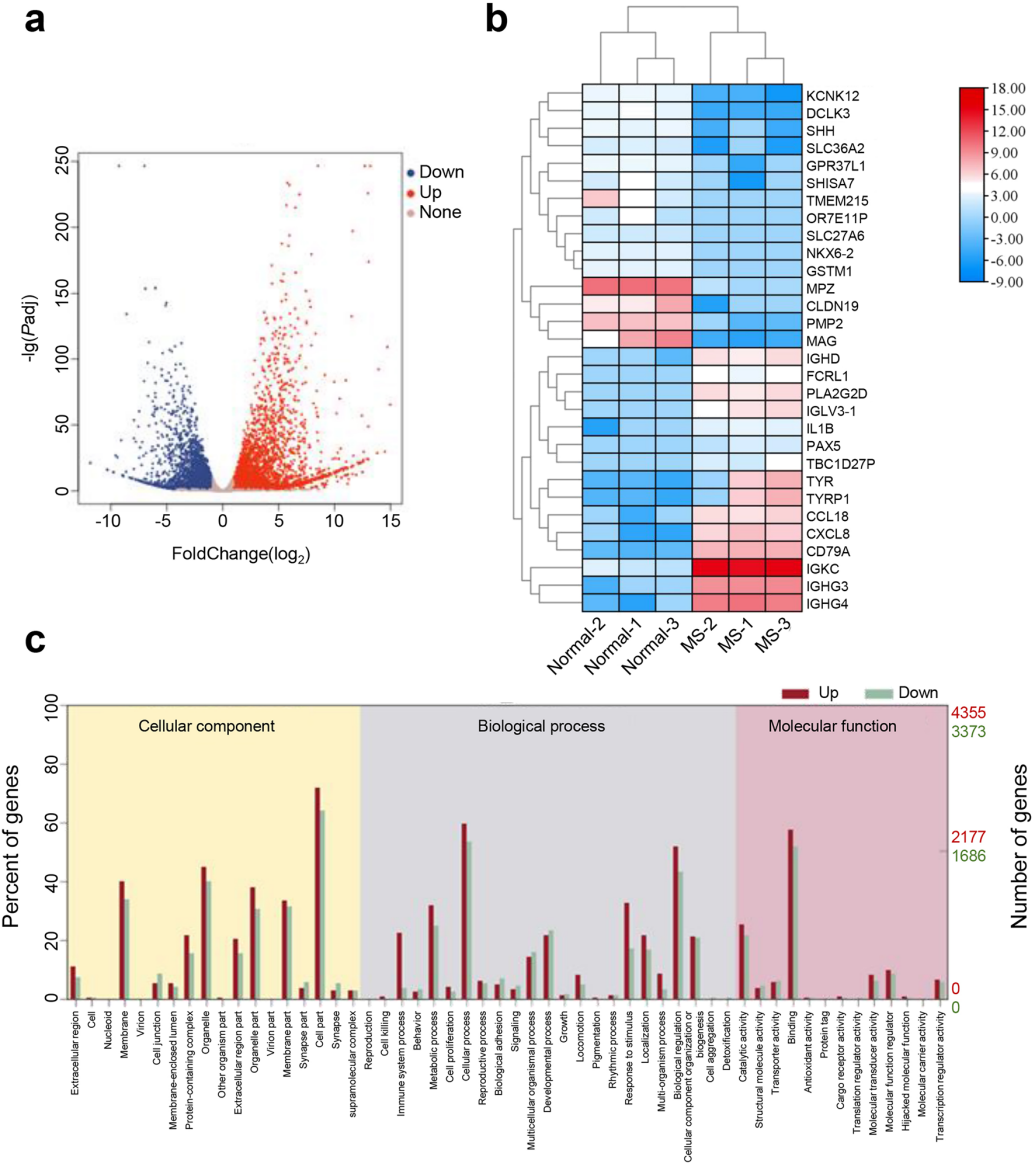
Transcriptomic analysis of MS. **a** Volcano plot showing the differentially expressed genes (DEGs) between MS and normal nerves. **b** Heatmap showing the 15 most significantly upregulated and downregulated genes. **c** KEGG enrichment analysis of DEGs showing the enriched signalling pathways in MS

Transcriptome sequencing identified a total of 7728 differentially expressed genes (DEGs) in MS tissues, including 4355 upregulated and 3373 downregulated genes (Fig. 3a). The 15 most significantly upregulated genes included immunoglobulin-related genes (*IGHG3*, *IGHG4*, *IGKC*, *IGLV3-1*, *IGHD*, *CD79A*, *FCRL1*), inflammatory factors (*IL1B*, *CXCL8*, *CCL18*), melanin synthesis-related genes (*TYR*, *TYRP1*) and a tumorigenesis-associated gene (*PAX5*), indicating robust immune responses and the involvement of melanin synthesis in MS pathogenesis. In contrast, the 15 most significantly downregulated genes were primarily nerve myelin sheath-related (*MAG*, *PMP2*, *MPZ*, *NKX6-2*), reflecting marked suppression of normal myelin sheath expression in MS (Fig. 3b). KEGG enrichment analysis further revealed that these DEGs were predominantly enriched in immune response and cell anabolism pathways (Fig. 3c).

### Proteomic Analysis of MS

**Fig. 4:**
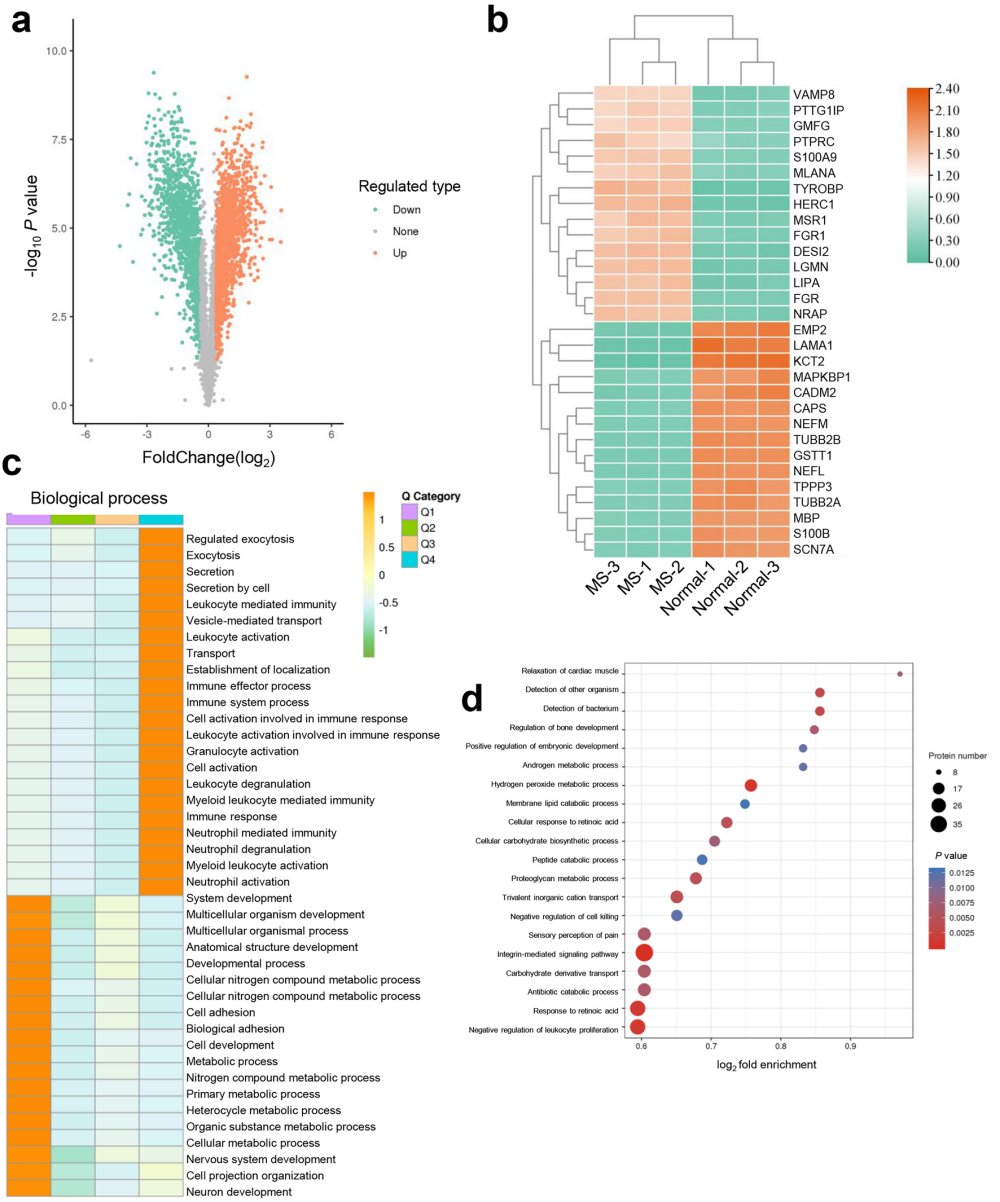
Proteomic analysis of MS. **a** Volcano plot showing the differentially expressed proteins between MS and normal nerves. **b** Heatmap showing the 15 most significantly upregulated and downregulated proteins. **c, d** Clustering analysis (**c**) and KEGG enrichment analysis (**d**) of the differentially expressed proteins showing the enriched signalling pathways in MS

A total of 2991 differentially expressed proteins, including 1730 upregulated proteins and 1261 downregulated proteins, were identified by proteomic analysis of MS samples (Fig. 4a). Further analysis of the 30 most significantly differentially expressed proteins revealed that the 15 most upregulated ones included mainly tumorigenesis-related proteins, such as PTTG1IP and LGMN; melanin synthesis-related proteins, such as MLANA, TYROBP, and FGR; and inflammatory response-related proteins, such as MSR1. Moreover, the expression of vesicle-associated membrane protein 8 (VAMP8) was also significantly upregulated in MS, indicating that extracellular vesicles are also involved in the development of MS (Fig. 4b). Genes encoding EMP2, LAMA1, KCT2, and other genes related to normal cell structure were significantly downregulated in MS. NEFM, NEFL, TUBB2B, TUBB2A, TPPP3, SCN7A, and MBP, key structural proteins of nerve fibres, were also significantly downregulated (Fig. 4b). Clustering and KEGG analyses revealed that the differentially expressed proteins were enriched primarily in pathways associated with specific biological processes, such as inflammatory immunity, immune regulation, and metabolism, suggesting that MS tumour cells drive these pathways to induce biological processes including tumorigenesis and tumour immunity (Fig. 4c, d).

### Activation of the Tyrosine Synthesis Pathway in MS

**Fig. 5:**
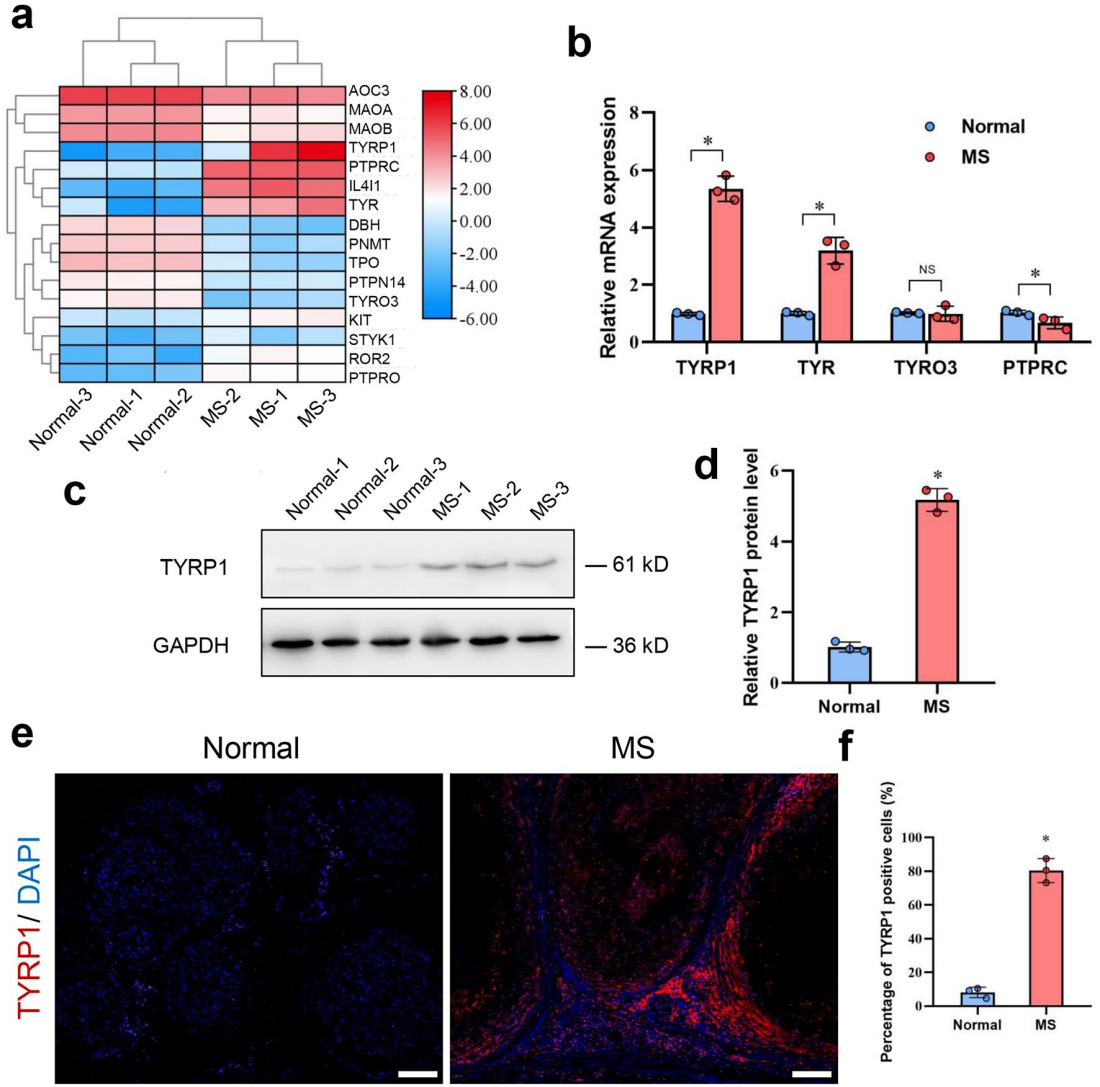
The expression pattern of TYRP1 in MS. **a** Heatmap showing the gene expression changes in the tyrosine synthesis pathway. **b** qRT-PCR analysis of the changes in TYRP1, TYR, TYRO3, and PTPRC expression between MS and normal nerves. **c**, **d** Western blot analysis (**c**) and statistical analysis (**d**) of the changes in TYRP1 expression between MS and normal nerves. **e** TYRP1 IF staining of MS and normal nerves. Scale bar = 100 μm. **f** Statistics of TYRP1^+^ cells in MS and normal nerves. The results are shown as the mean ± SD; ^*^P < 0.05 vs. normal nerves

KEGG analysis of the transcriptome sequencing results revealed abnormal activation of the tyrosine synthesis pathway in MS (Fig. S1). The DEGs in the tyrosine synthesis pathway between MS and normal nerves were subsequently analysed, and the results revealed that most genes, including *TYRP1*, *PTPRC*, *IL4I1*, *TYR*, *KIT*, *ROR2*, *PTPRO*, and others, were upregulated in the tyrosine synthesis pathway (Fig. 5a). A qRT-PCR was subsequently used to verify the changes in the expression patterns of these DEGs in MS. The results revealed that the expression of *TYRP1* and *TYR* was significantly increased in the MS group compared with the normal group (*P* < 0.05), with *TYRP1* showing the greatest increase (Fig. 5b). Changes in the protein expression of TYRP1 were subsequently verified using Western blotting, and the results revealed that the protein expression of TYRP1 was also significantly increased in MS (Fig. 5c, d). In addition, IF staining of TYRP1 revealed that TYRP1 was almost undetectable in normal nerves. In MS, however, TYRP1 was overexpressed in cells within the perineurium and between nerve bundles (Fig. 5e). Combined with the IHC staining results described above, these cells are highly consistent with MS cells. Further statistical analysis revealed that the number of TYRP1-positive cells in the MS group was significantly greater than that in the normal group (Fig. 5f).

### Activation of MS in the TRP-Mediated Inflammatory Pathway

**Fig. 6:**
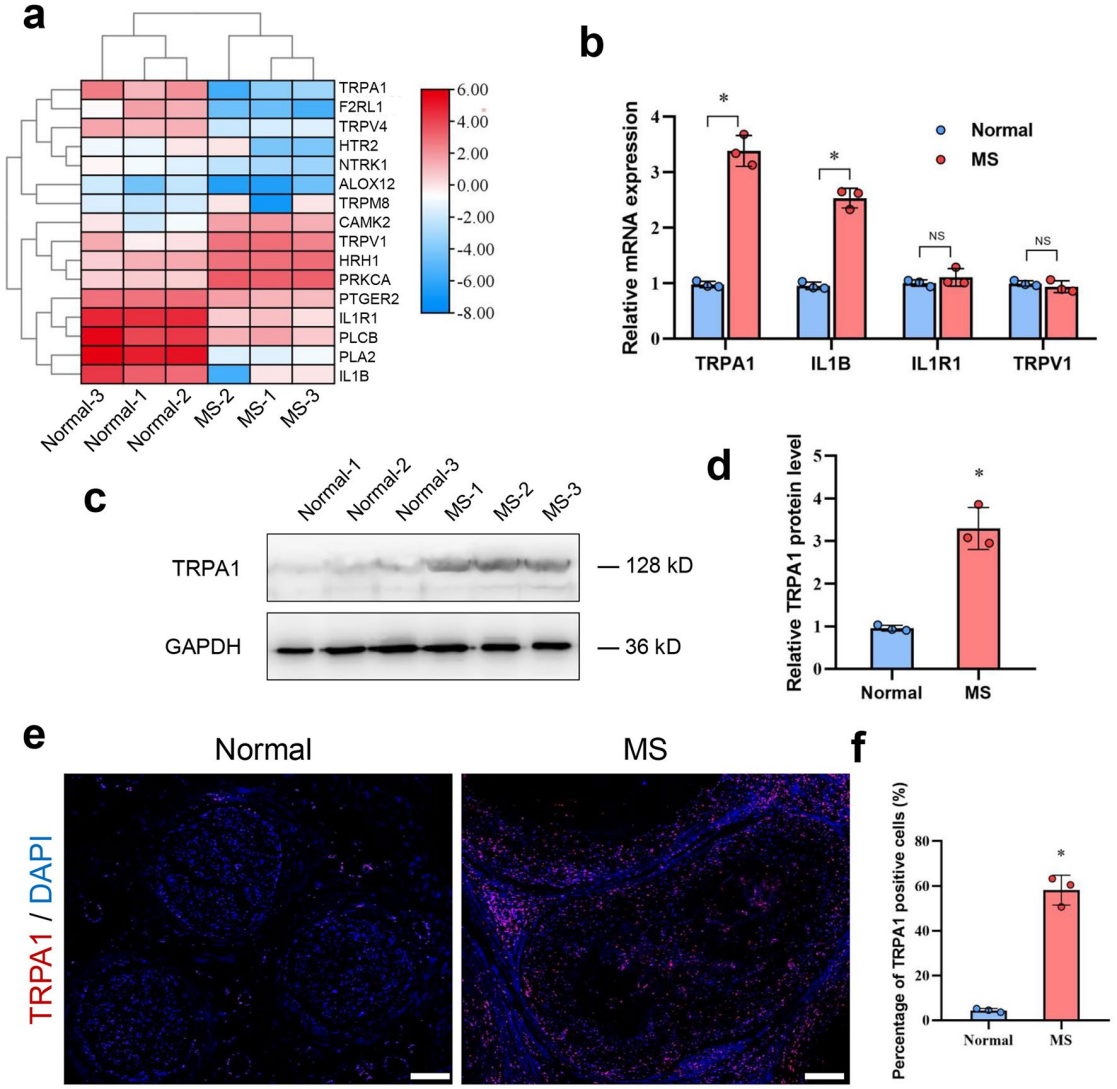
The expression pattern of TRPA1 in MS. **a** Heatmap showing the gene expression changes in the TRP-channel pathway. **b** qRT-PCR analysis of the changes in TRPA1, IL1B, IL1R, and TRPV1 expression between MS and normal nerves. **c**, **d** Western blot analysis (**c**) and statistical analysis (**d**) of the changes in TRPA1 expression between MS and normal nerves. **e** TRPA1 IF staining of MS and normal nerves. Scale bar = 100 μm. **f** Statistics of TRPA1^+^ cells in MS and normal nerves. The results are shown as the mean ± SD. ^*^P < 0.05 vs. normal nerves

Moreover, KEGG analysis of the transcriptome sequencing results also revealed that TRP channel-related inflammatory factors were abnormally activated in MS (Fig. S2). Differences in the expression of TRP channel-related inflammatory factors, such as *TRPV1*, *TRPA1*, *IL1B*, and *IL1R1*, were subsequently analysed, and the results revealed that, among the TRP channel-related inflammatory factors, some genes, such as *TRPV1*, *TRPA1*, and *IL1B*, were upregulated (Fig. 6a). A qRT-PCR assay was subsequently used to verify the changes in the expression patterns of these DEGs in MS. The results revealed that the expression levels of *TRPA1* and *IL1B* were significantly greater in the MS group than in the normal group, with that of *TRPA1* showing the greatest increase (Fig. 6b). Subsequent Western blotting was used to confirm the changes in the expression of the TRPA1 protein, and the results revealed that the expression of the TRPA1 protein was also significantly increased in MS (Fig. 6c, d). In addition, IF staining of TRPA1 revealed that TRPA1 was only weakly expressed in normal nerves but was overexpressed around tumour cells within the perineurium and between nerve bundles in MS (Fig. 6e). Further statistical analysis revealed that the number of TRPA1-positive cells in the MS group was significantly greater than that in the normal group (Fig. 6f).

### BTK as a Drug Target for the Treatment of MS

**Fig. 7:**
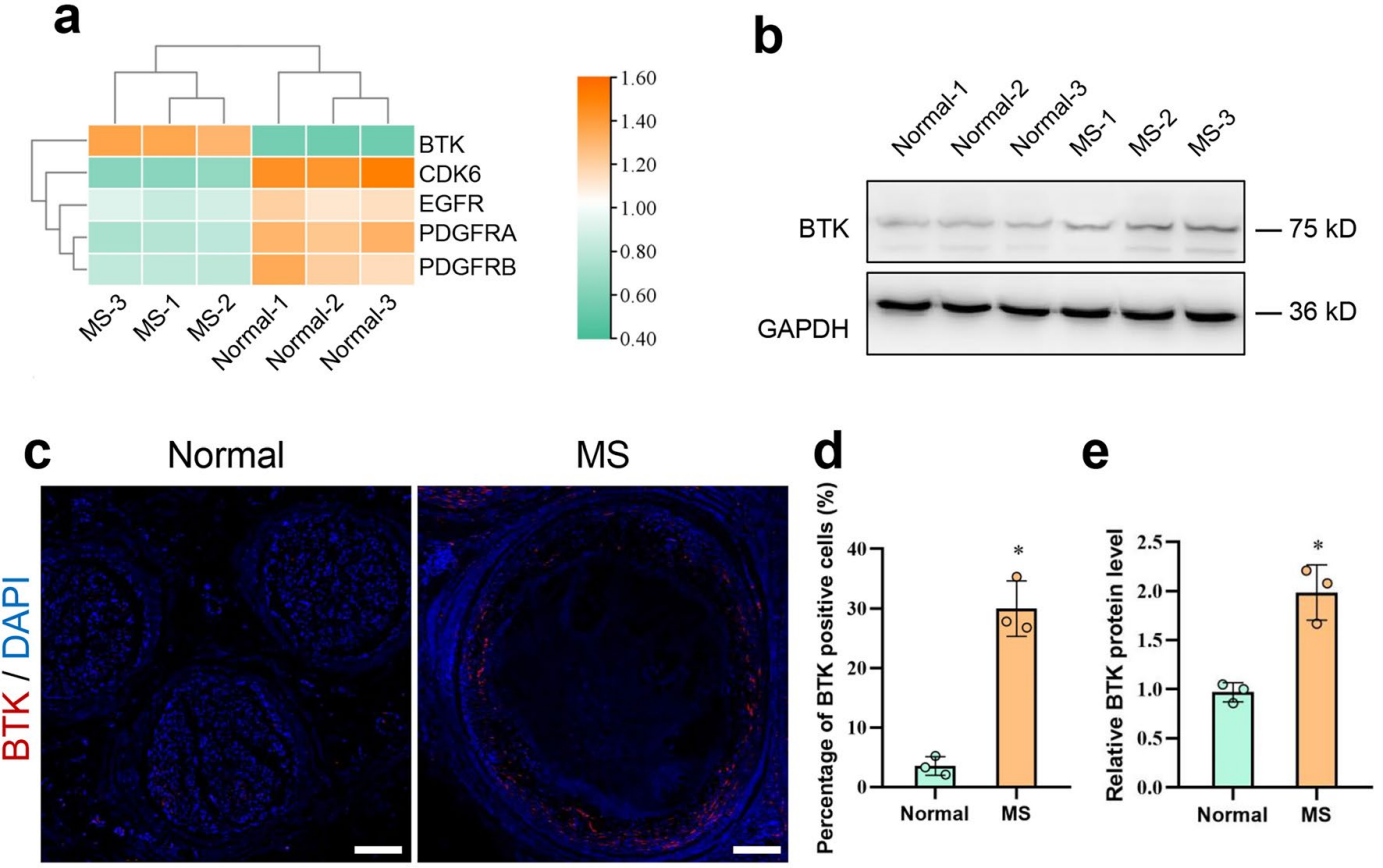
The expression pattern of BTK in MS. **a** Heatmap showing the expression of clinically actionable drug targets. **b**, **e** Western blot analysis (**b**) and statistical analysis (**e**) of the changes in BTK expression between MS and normal nerves. **c** BTK IF staining of MS and normal nerves. Scale bar = 100 μm. **d** Statistics of BTK^+^ cells in MS and normal nerves. The results are shown as the mean ± SD; ^*^P < 0.05 vs. normal nerves

Molecular targeted drugs show superior efficacy to conventional radiotherapy and chemotherapy for most malignant tumours, but targeted therapy for malignant MS remains unreported, leading to poor patient prognosis. Based on proteomic data, the expression of FDA-approved molecular targeted drug targets in MS tissues was analysed and the results showed that only BTK, EGFR, CDK6, PDGFRA and PDGFRB were expressed in both MS and normal groups, with only BTK significantly upregulated in MS (Fig. 7a). Western blotting and IF assays validated these results (Fig. 7b, c), confirming markedly higher BTK expression and ~ 30% more BTK-positive cells in MS tissues relative to normal controls (Fig. 7d, e). These findings indicate that BTK inhibitors (e.g., zanubrutinib, ibrutinib) may have therapeutic potential for malignant MS, providing novel insights for its clinical management.

## Discussion

MS is a rare nerve tumor with an incidence rate of approximately 0.01% [13, 14]. Notably, the number of MS case report has gradually increased in recent years, which suggests a potential rise in its incidence [15–18]. To date, the pathogenesis of MS remains poorly elucidated, resulting in a lack of specific therapeutic targets for clinical intervention. In this study, we elucidated the molecular characteristics and growth pattern of MS via integrated histologic, transcriptomic and proteomic analyses, identified core pathogenic pathways associated with its tumorigenesis and neuropathic pain, and verified BTK as a potential molecular target for treatment, providing novel experimental evidence for the clinical management of this rare tumour.

**Fig. 8:**
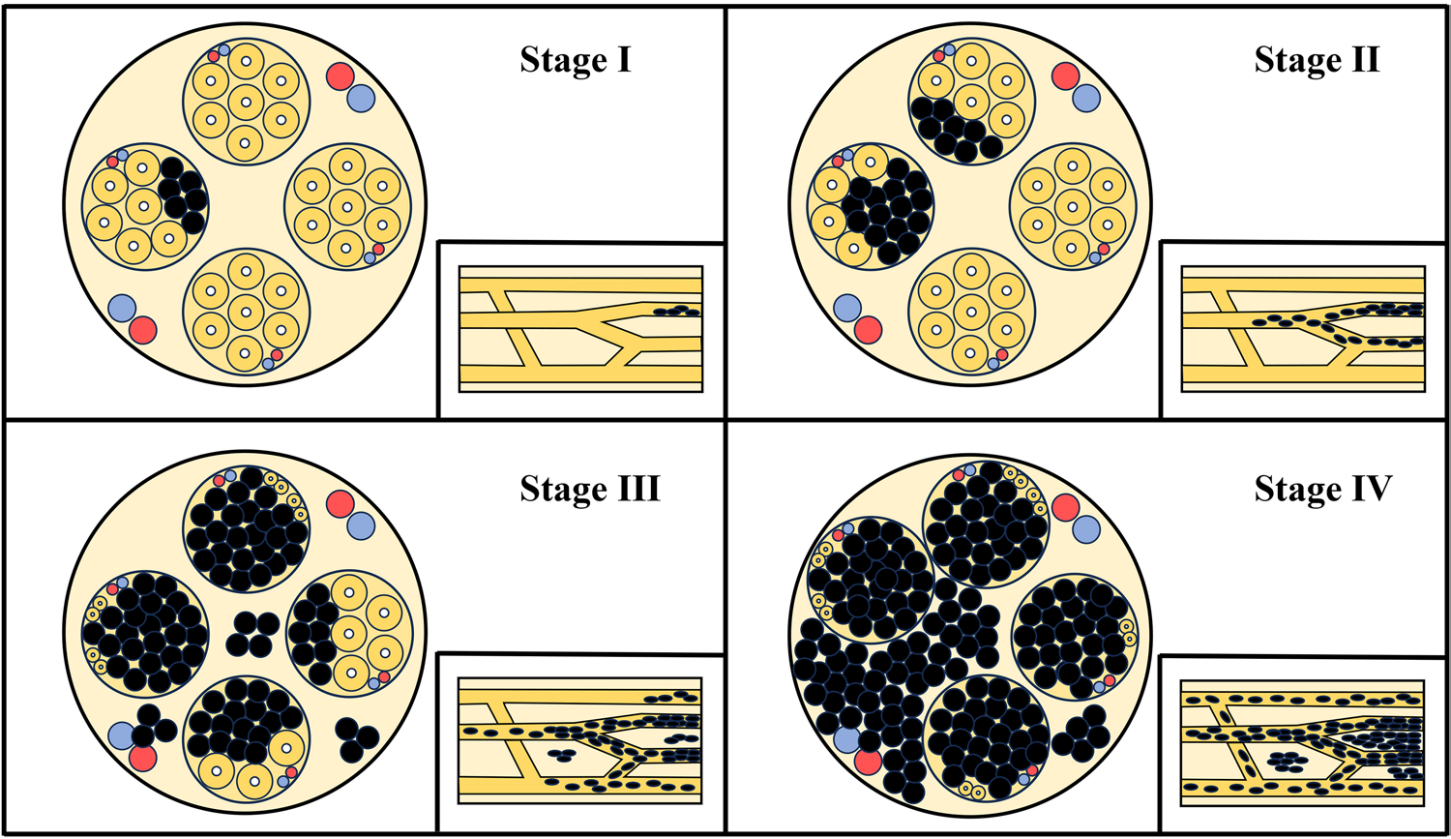
Schematic illustration of the MS growth pattern

Histologic analysis defined the typical growth pattern of MS (Fig. 8). Initially, individual Schwann cells in nerve bundles undergo malignant transformation into MS cells via abnormally activated tyrosine kinase pathways and tumour-related factors, proliferating and spreading within the perineurial space with no obvious neurological symptoms due to limited tumor burden. As tumour cells expand, the primary perineurium becomes filled with MS cells that spread along the nerve; the perineurium acts as a basement membrane to prevent direct invasion, while epineurial inter-bundle communicating branches enable MS cell migration to adjacent nerve bundles without perineurial breach. Tumour cells can also invade the intraneural vascular network, leading to local metastasis. Perineurial tumor overgrowth compresses nerve fibres, inducing paresthesia and muscle weakness. At this stage, MS cells colonize epineurial connective tissue vasculature—where wider space allows extensive proliferation, with the epineurium still restricting invasion—and gain access to larger blood vessels, increasing the risk of distant haematogenous metastasis. Ultimately, unregulated tumour proliferation elevates intra-nerve pressure under perineurial restraint, causing severe nerve fibre compression, structural disruption and peripheral displacement of residual fibres. Central bundle cells (tumor and normal) undergo necrosis due to overcrowding and nutrient deficiency; the affected nerve loses full function, and enlarged distant metastases impair organ function, drastically reducing patient survival.

At the molecular level, MS pathogenesis has been rarely investigated by multi-omics analysis, with only a few studies linking it to genetic mutations and specific gene overexpression [13, 19–23]. Approximately 18% of Carney complex patients (caused by *PRKAR1A* gene deletion) develop MS, and overexpression of melanin synthesis-related genes (*HMB45*, *TYR*) and tumor-related gene *SMARCB1* has been observed in MS tissues [13, 19–25]. Consistent with these findings, our transcriptomic and proteomic analyses revealed abnormal upregulation of multiple genes in the tyrosine synthesis pathway in MS, and we for the first time confirmed significant TYRP1 overexpression in MS tissues via IF staining, Western blotting and qRT-PCR. As a key catalytic enzyme in melanin synthesis, TYRP1 overexpression (together with TYR upregulation) leads to a melanin-rich state in MS tumour cells; aberrant TYRP1 activation drives tumour proliferation pathways, and its targeted inhibition has improved melanoma patient survival, providing indirect evidence for its pro-tumorigenic role in MS [26–29]. These results confirm that abnormal activation of the tyrosine synthesis pathway, especially TYRP1 overexpression, is a core molecular feature underlying MS tumorigenesis and its melanin-producing characteristic.

Severe refractory neuropathic pain is a prominent clinical manifestation of MS, yet its mechanism has long remained unclear [30–32]. In the present study, integrated transcriptomic and proteomic analyses revealed abnormal activation of the TRP channel-related inflammatory pathway in MS tissues. In addition to TRP family members, inflammatory factors including IL-1β were also found to be upregulated at the omics level, suggesting that inflammatory processes may also contribute to MS pathogenesis and symptom development. Among the altered molecules, TRPA1 was selected for further verification, and its significant upregulation in MS was confirmed at both mRNA and protein levels by qRT-PCR, Western blotting and IF staining. As a key ion channel involved in nociception and neuroinflammation, TRPA1 can be activated by multiple stimuli and further promotes the release of pro-inflammatory factors [33–36]. Of note, IL-1β, a classic pro-inflammatory cytokine, has been widely reported to interact with TRPA1: IL-1β can enhance the expression and activity of TRPA1, thereby amplifying neuronal excitability and pain signaling; conversely, activated TRPA1 may in turn promote the maturation and release of IL-1β, forming a positive feedback loop that aggravates neuroinflammation and pain [37–41]. Although the expression and localization of IL-1β were not further validated experimentally in this study, the combined omics results and previous literature strongly suggest that the interaction between TRPA1 and IL-1β may play an important role in MS-associated pain. Together with the morphological observation that tumor proliferation leads to obvious compression of nerve fibres, we propose that the intractable pain in MS is mediated by TRPA1 overexpression, IL-1β-related inflammatory activation, and mechanical nerve compression. These findings indicate that TRPA1 may serve as a promising target for the relief of neuropathic pain in patients with MS.

Traditionally considered a benign tumour with ~ 10% malignant transformation rate, MS is now recognized as a malignant neoplasm due to its 13%–26% metastatic rate and frequent recurrence and distant metastasis [13, 42, 43]; conventional radiotherapy and chemotherapy show limited efficacy, leading to poor prognosis for malignant MS [25, 44–46]. Studies have shown that among melanoma patients, a Ki67 rate > 10% indicates a poor prognosis, while a rate > 30% suggests an extremely high degree of malignancy [47, 48]. Our finding revealed a 16.67% Ki67 positivity rate in MS tissues—an intermediate-to-high level indicative of active proliferation, elevated malignancy and increased recurrence risk. Based on proteomic profiling, we screened FDA-approved molecular targeted drug targets in MS and identified BTK as the only target significantly upregulated in MS tissues. As a key tyrosine kinase in B-cell receptor signalling, BTK inhibitors effectively inhibit malignant B-cell lymphoma proliferation [49, 50], and BTK also modulates melanin synthesis—linking it to the abnormally activated tyrosine synthesis pathway in MS [51–53]. Studies have shown that third-generation BTK inhibitors (e.g., pirtobrutinib) exhibit superior safety and efficacy vs. first-generation agents (ibrutinib, zanubrutinib) [54]. Our tissue-level verification of significant BTK overexpression in MS is the first to identify BTK as a potential therapeutic target for MS, and BTK inhibitors may treat MS by inhibiting tumor proliferation and modulating the abnormal activation of the melanin synthesis pathway.

Nevertheless, this study has several limitations. First, the small sample size—due to the extreme rarity of MS—may limit the generalizability of our results, and future studies with expanded cohorts are needed for validation. Second, while we identified core pathogenic pathways (tyrosine synthesis, TRP-mediated inflammation) and the BTK target, their specific molecular regulatory mechanisms in MS tumorigenesis and pain development remain to be elucidated by functional experiments. Additionally, transcriptomic and proteomic result discrepancies may be attributed to post-transcriptional/translational modifications, and further research is required to clarify these regulatory processes. Finally, the in vivo and clinical efficacy of BTK inhibitors in MS needs verification via preclinical animal experiments and prospective clinical trials.

## Conclusions

This study systematically revealed the histomorphological growth pattern, transcriptomic and proteomic characteristics of MS. We identified that abnormal activation of the tyrosine synthesis pathway and upregulation of TYRP1 are closely associated with the melanin production feature of MS. In addition, the TRPA1-mediated neuroinflammatory pathway and mechanical nerve compression jointly contribute to severe neuropathic pain in patients with MS. Furthermore, we first reported that BTK is significantly overexpressed in MS and may serve as a novel potential therapeutic target for this rare tumor. Collectively, our findings provide new insights into the pathogenesis, clinical symptom mechanism and targeted therapy of MS.

## Supplementary Information

Below is the link to the electronic supplementary material.

Supplementary file1 (PDF 148 KB)

Supplementary file2 (DOCX 148 KB)

## Data Availability

The raw RNA sequencing data have been submitted to the SRA database, and the BioProject accession number is PRJNA1083972. The mass spectrometry proteomics data have been deposited to the ProteomeXchange Consortium (https://proteomecentral.proteomexchange.org) via the iProX partner repository with the dataset identifier PXD050541.
