# Supplementary material for: Decoding the Histomorphological and Multi-Omic Characteristics of Melanotic Schwannoma
Source: Curr Med Sci. 2026 Apr 17;46(3):690–703. doi: 10.1007/s11596-026-00197-6 (PMC13314831; doi:10.1007/s11596-026-00197-6)

The original blots figures

Fig.5c

TRPA1:

Normal-1, Normal-2, Normal-3, MS-1, MS-2, MS-3

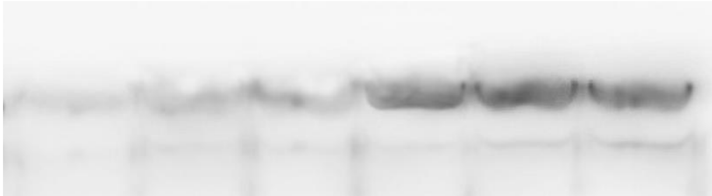

GAPDH:

Normal-1, Normal-2, Normal-3, MS-1, MS-2, MS-3

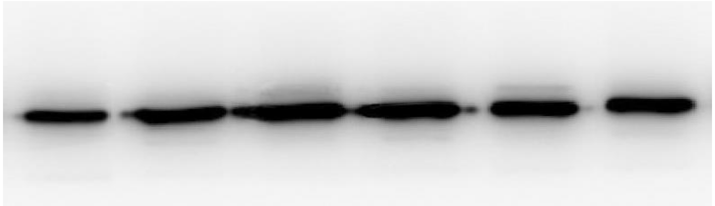

Fig.6c

TYRP1:
Normal-1, Normal-2, Normal-3, MS-1, MS-2, MS-3

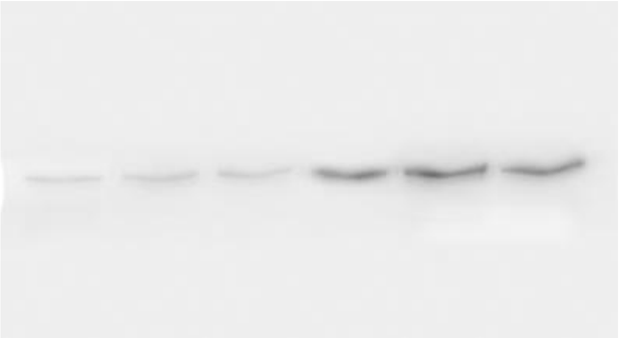

GAPDH:
Normal-1, Normal-2, Normal-3, MS-1, MS-2, MS-3

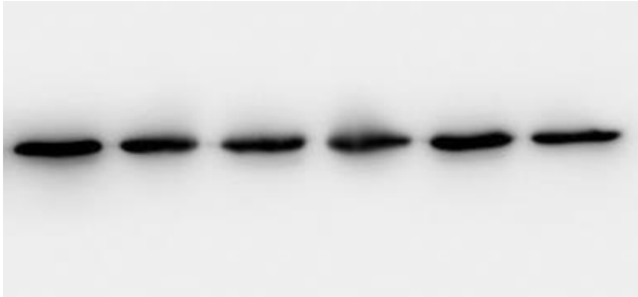

Fig.7b

BTK:

Normal-1, Normal-2, Normal-3, MS-1, MS-2, MS-3

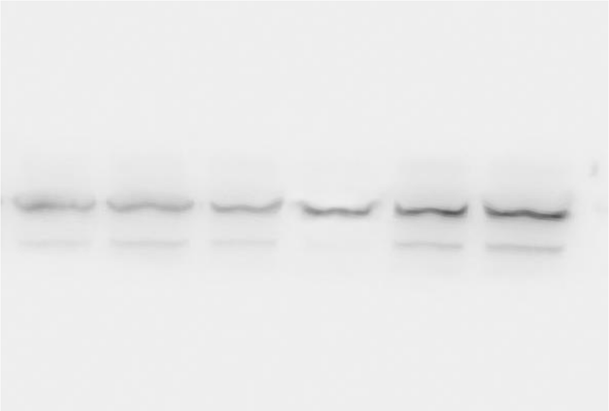

GAPDH:

Normal-1, Normal-2, Normal-3, MS-1, MS-2, MS-3

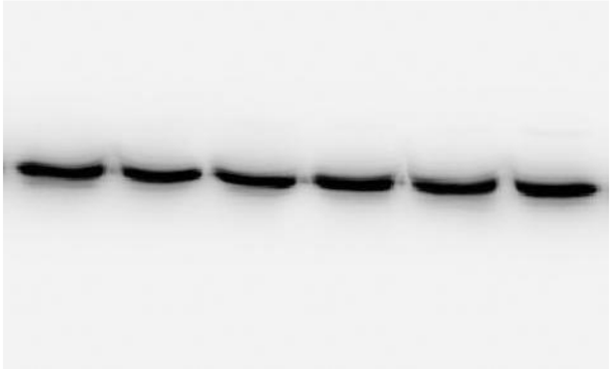

Supplement: Supplementary file 1 — Supplementary file1 (PDF 148 KB) [file 11596_2026_197_MOESM1_ESM.pdf]
