# Supplementary material for: Decoding the Histomorphological and Multi-Omic Characteristics of Melanotic Schwannoma
Source: Curr Med Sci. 2026 Apr 17;46(3):690–703. doi: 10.1007/s11596-026-00197-6 (PMC13314831; doi:10.1007/s11596-026-00197-6)
Supplement: Supplementary file 2 — Supplementary file2 (DOCX 148 KB) [file 11596_2026_197_MOESM2_ESM.docx]

**Supplementary Materials and Methods**

**1 Sample Collection**

Tumor tissue samples of multiple sclerosis (MS) were obtained from three MS patients who underwent surgical treatment at our hospital. Normal nerve tissue samples (n = 3) for the control group were collected from patients who received amputation surgery due to limb traumatic deformity at our hospital. Immediately after resection, the surrounding non-target tissues of the samples were carefully dissected and removed, followed by three rinses with phosphate-buffered saline (PBS). The samples were then blotted dry and stored in liquid nitrogen or 4% paraformaldehyde for subsequent experimental detection.

**Patient Information of the Study Samples**

| Sample | Sex | Age | Sampling site |
| --- | --- | --- | --- |
| MS1 | Male | 62 | Median nerve of left forearm |
| MS2 | Male | 65 | Left brachial plexus |
| MS3 | Male | 43 | Right digital nerve |
| Control1 | Male | 52 | Tibial nerve of right ankle |
| Control2 | Male | 58 | Tibial nerve of left ankle |
| Control3 | Male | 34 | Left digital nerve |

**2 Histomorphometric Measurement**

We performed quantitative analysis of histological and immunostained images of nerve fibers using histomorphometric methods. For each group, 20 high-power fields were randomly selected from the stained images of the three samples, and relevant parameters were counted for each field, including nerve fiber diameter, nerve fiber aspect ratio, and nerve fiber density. Statistical comparisons between groups were then conducted based on these data.

**Table S1 Antibody information**

| Protein Name | Company | Product code | Dilution ratio |
| --- | --- | --- | --- |
| S100 | Servicebio (CN) | GB11397 | 1:100 |
| Ki67 | Servicebio (CN) | GB111499 | 1:100 |
| β3-Tubulin | Servicebio (CN) | GB12139 | 1:100 |
| TYRP1 | Invitrogen (USA) | PA5-107304 | 1:100 (IF); 1:500 (WB) |
| TRPA1 | Invitrogen (USA) | PA5-109320 | 1:100 (IF); 1:500 (WB) |
| BTK | Proteintech (USA) | 21581-1-AP | 1:100 (IF); 1:500 (WB) |

**Table S2 Primer information**

| Gene name | Forward primer (5'–3') | Reverse primer (5'–3') |
| --- | --- | --- |
| TYRP1 | TCTCTGGGCTGTATCTTCTTCC | GTCTGGGCAACACATACCACT |
| TYR | GCAAAGCATACCATCAGCTCA | GCAGTGCATCCATTGACACAT |
| TYRO3 | CGGTAGAAGGTGTGCCATTTT | CGATCTTCGTAGTTCCTCTCCAC |
| PTPRC | ATTACCTGGAATCCCCCTCAAA | TTGTGAAATGACACATTGCAGC |
| TRPA1 | TGTGACGATATGGACACCTTCT | TTGAAGTTTCGGAGATTTGGGTT |
| IL1B | AGCTACGAATCTCCGACCAC | CGTTATCCCATGTGTCGAAGAA |
| IL1R1 | ATGAAATTGATGTTCGTCCCTGT | ACCACGCAATAGTAATGTCCTG |
| TRPV1 | CAGGCTCTATGATCGCAGGAG | TTTGAACTCGTTGTCTGTGAGG |


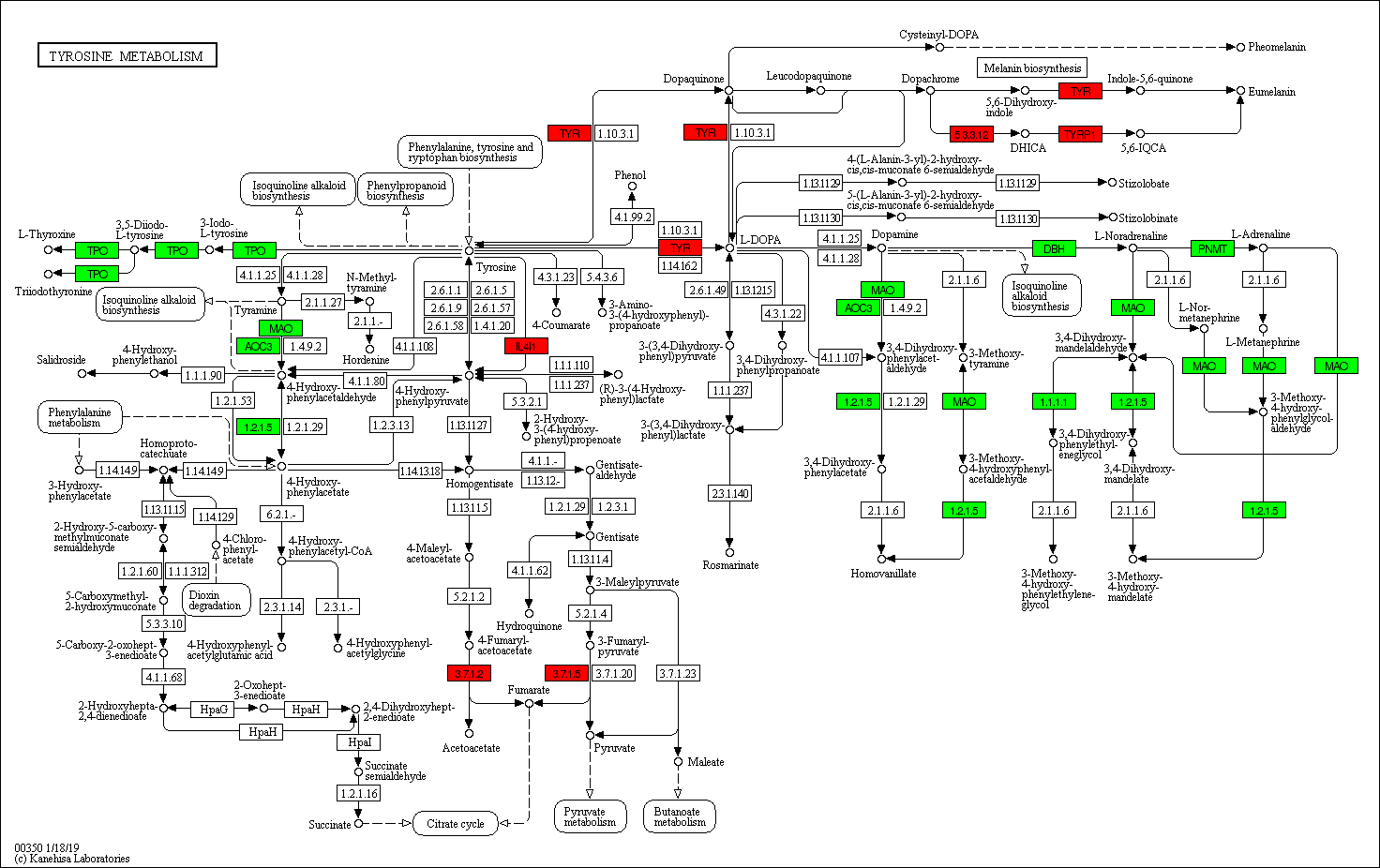


**Fig. S1 KEGG map showing the DEGs of MS in tyrosine metabolism pathway**


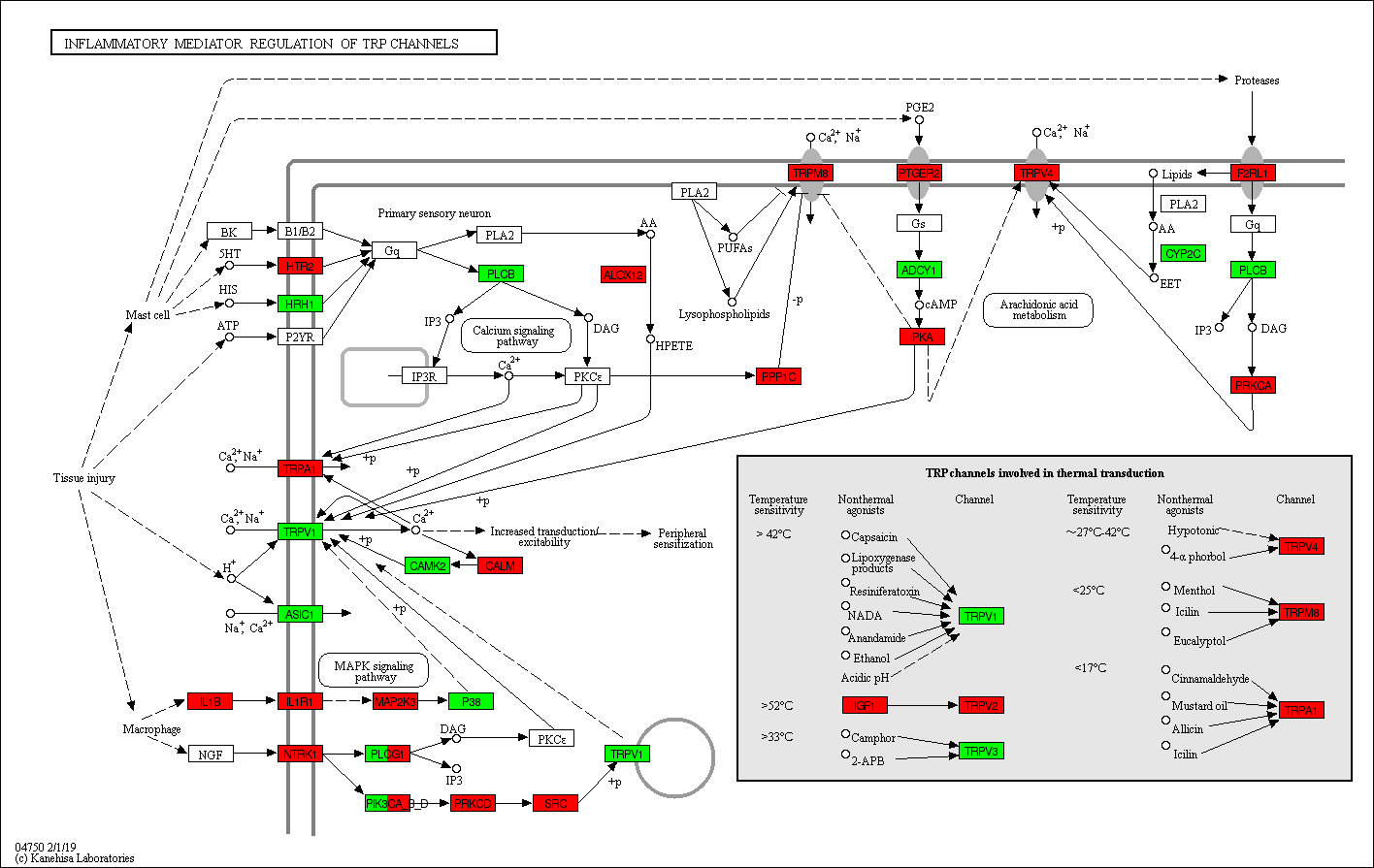


**Fig. S2 KEGG map showing the DEGs of MS in TRP channels pathway**
